# Supplementary material for: The Effects of Drying Techniques on Phytochemical Contents and Biological Activities on Selected Bamboo Leaves
Source: Molecules. 2022 Sep 30;27(19):6458. doi: 10.3390/molecules27196458 (PMC9571890; doi:10.3390/molecules27196458)
Supplement: Supplementary file 1 [file molecules-27-06458-s001.zip › molecules-1910761-supplementary.pdf]

Supplementary Materials

The Effects of Drying Techniques on Phytochemical Contents and Biological Activities on Selected Bamboo Leaves

Table of Contents

		Pages
**Figure S1.**	The standard curve of gallic acid for TPC	2
**Figure S2.**	The standard curve of quercetin for TFC	2
**Figure S3.**	The standard curve of Trolox for DPPH	3
**Figure S4.**	The standard curve of Trolox for ABTS	3
**Figure S5.**	The standard curve of Trolox for FRAP	4
**Figure S6.**	The mass spectrum of *Bambusa multiplex* (A) and caffeine (B)	5
**Figure S7.**	The mass spectrum of *Bambusa tuldoides* (A) and sparteine (B)	6
**Figure S8.**	The mass spectrum of *Bambusa tuldoides* (A) and papaverine (B)	7
**Figure S9.**	The mass spectrum of *Bambusa vulgaris* (A) and papaverine (B)	8
**Figure S10.**	The mass spectrum of *Dinochloa sublaevigata* (A) and papaverine (B)	9
**Figure S11.**	The mass spectrum of *Gigantochloa levis* (A) and papaverine (B)	10

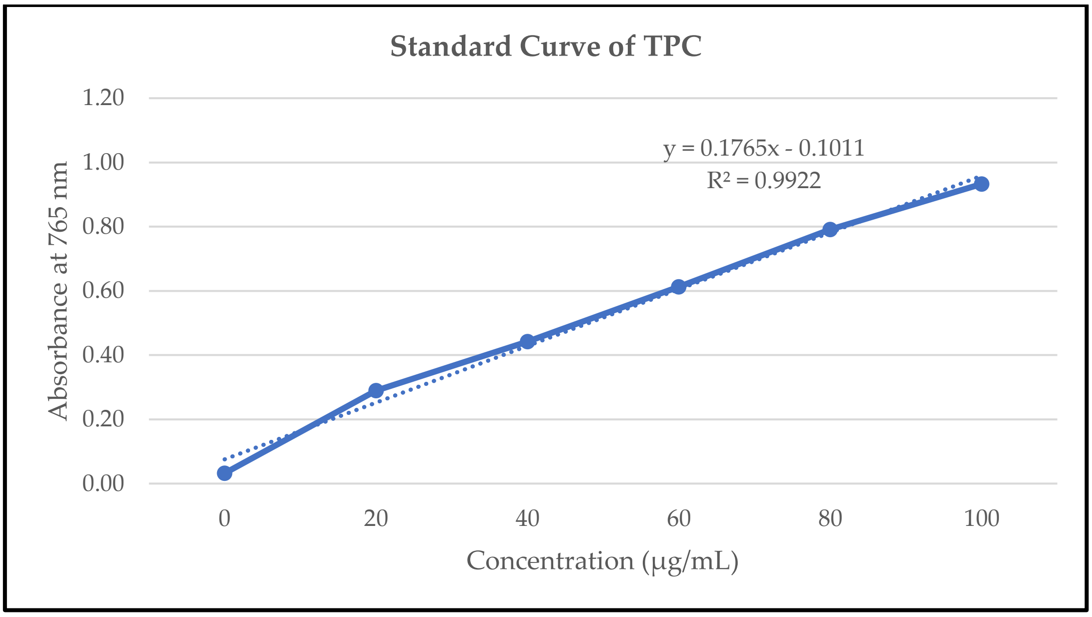

Figure S1. The standard curve of gallic acid for TPC.

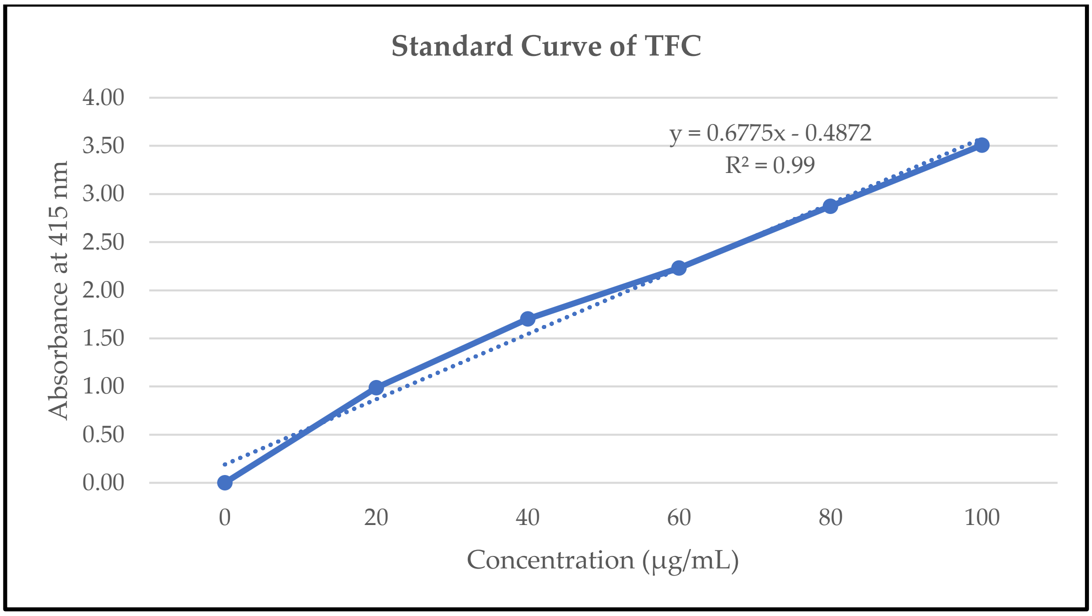

Figure S2. The standard curve of quercetin for TFC.

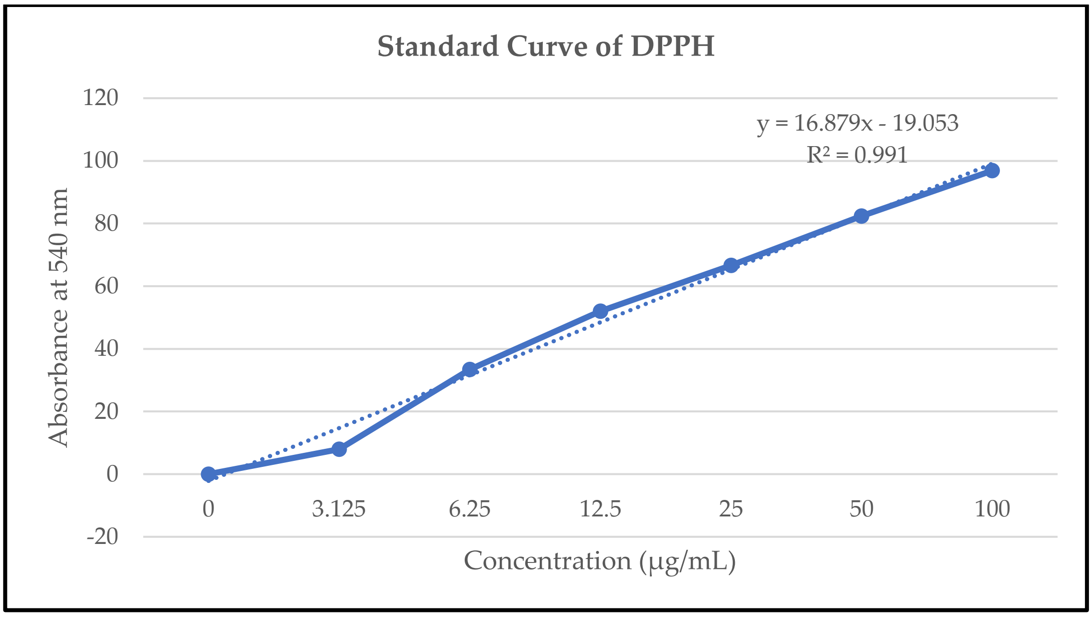

Figure S3. The standard curve of Trolox for DPPH.

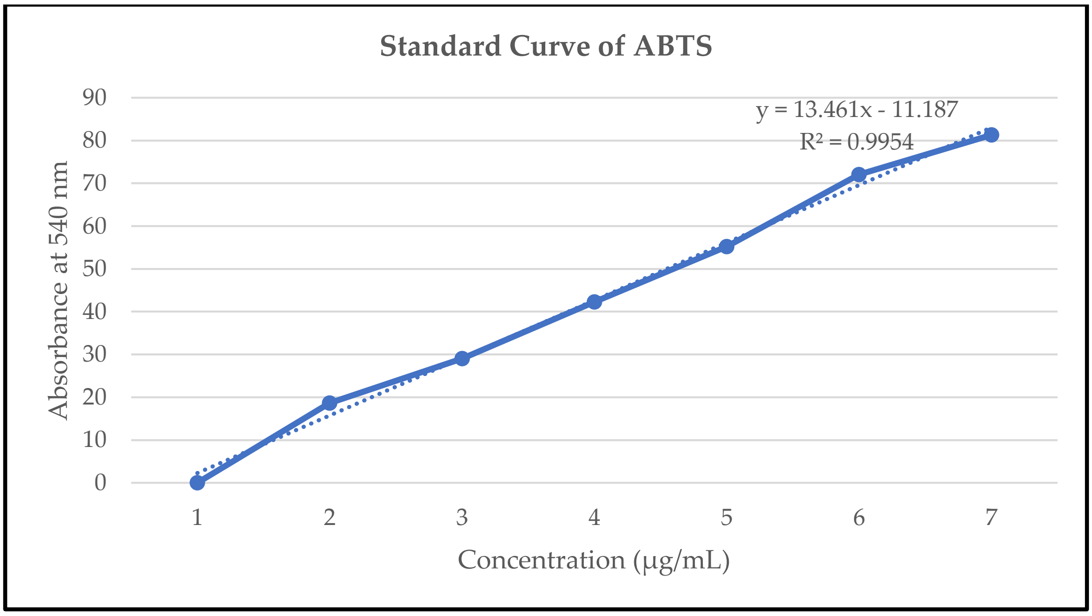

Figure S4. The standard curve of Trolox for ABTS.

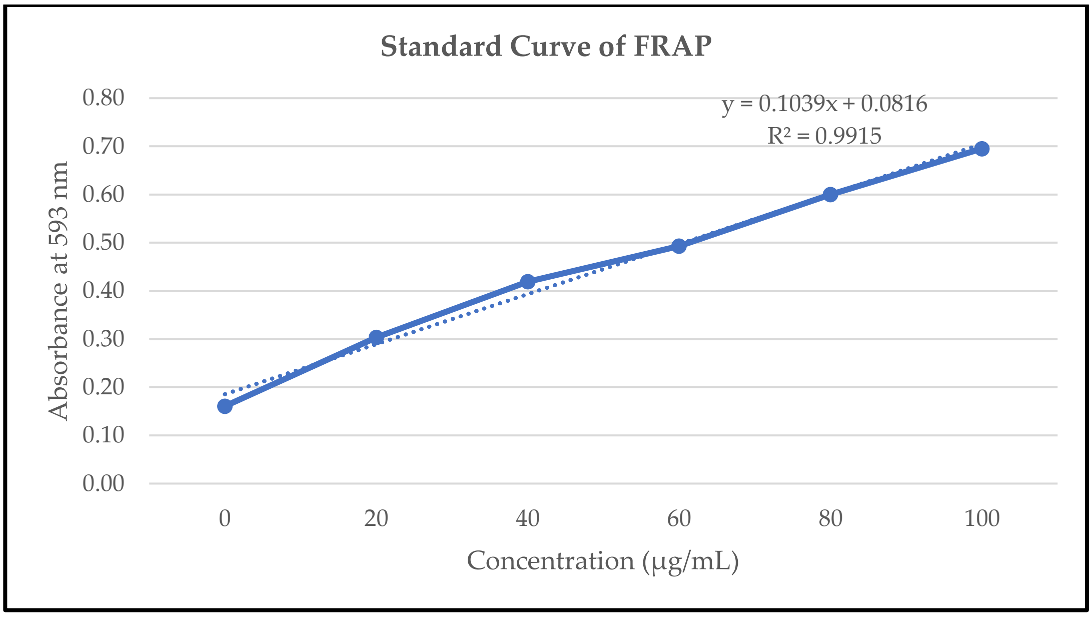

Figure S5. The standard curve of Trolox for FRAP.

A

B

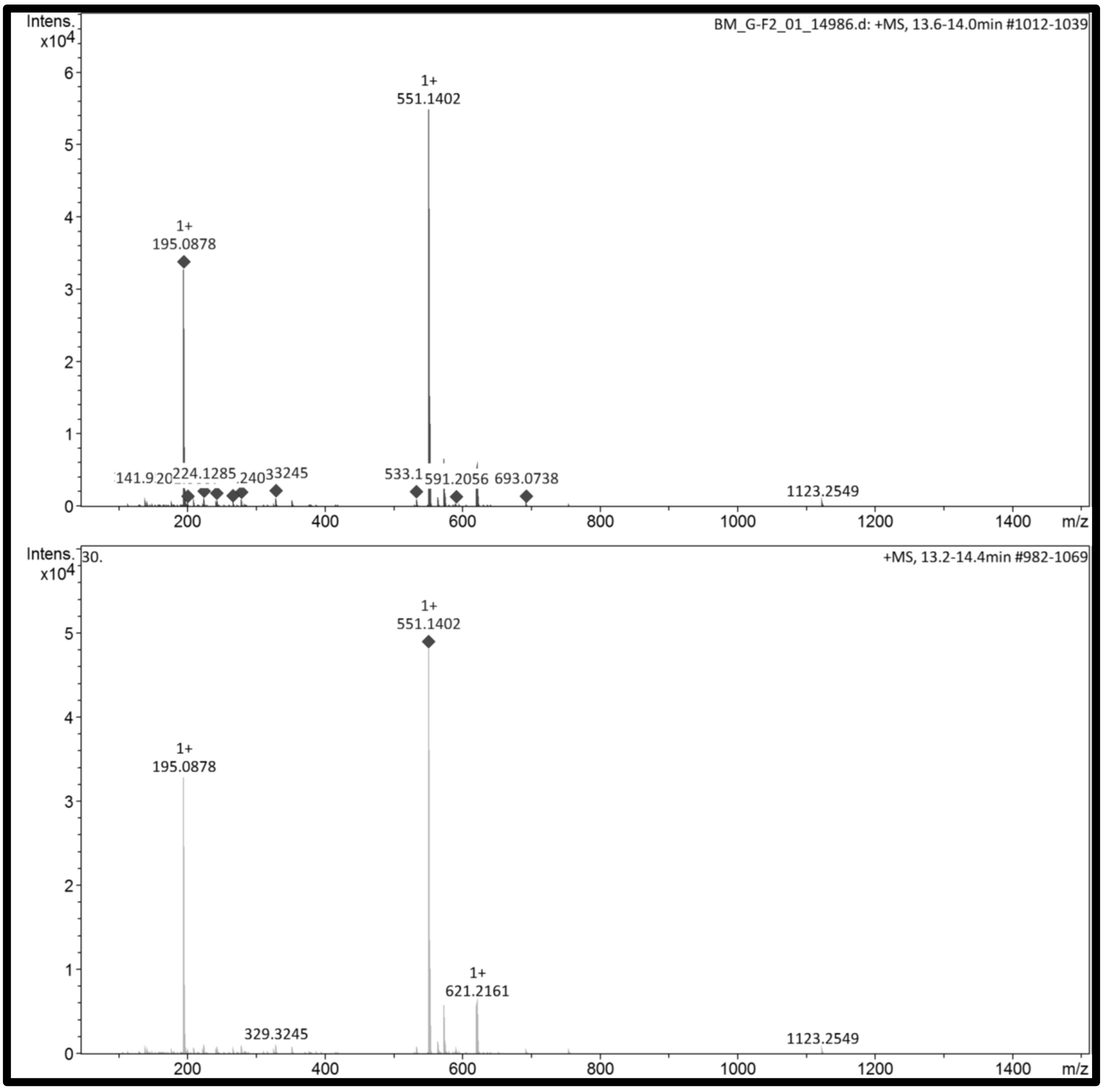

Figure S6. The mass spectrum of *Bambusa multiplex* (A) and caffeine (B).

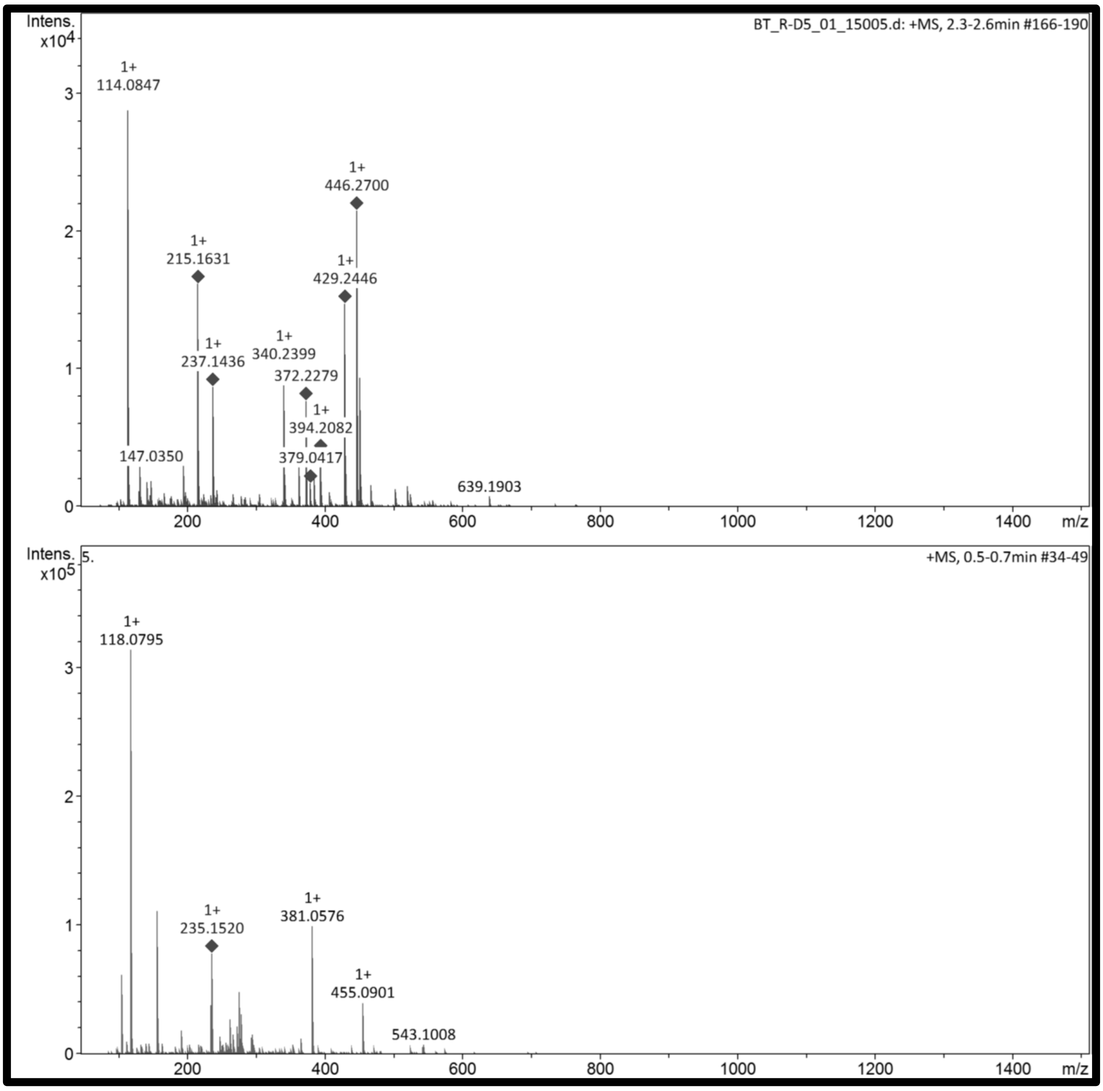

Figure S7. The mass spectrum of *Bambusa tuldoides* (A) and sparteine (B).

A

B

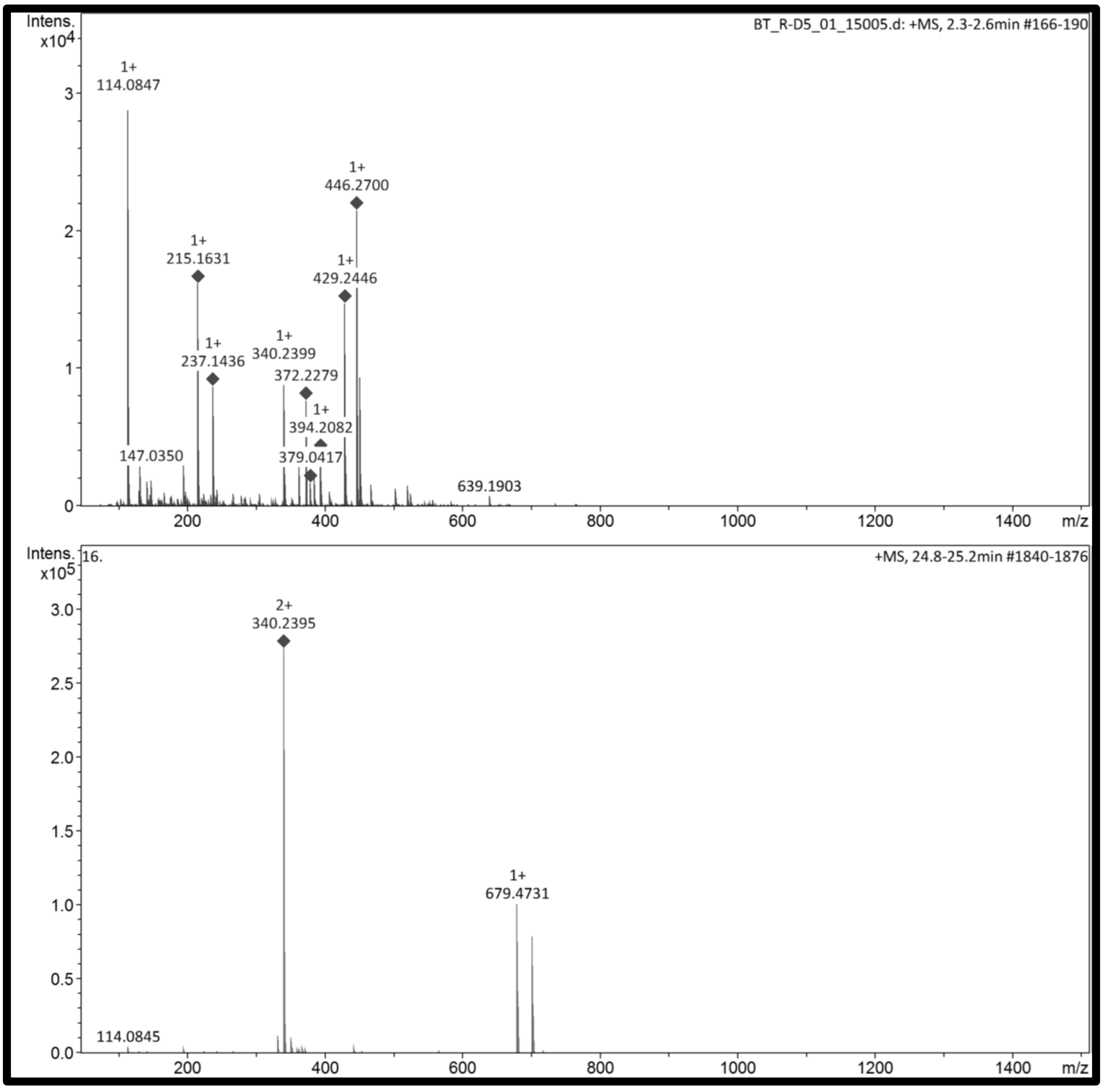

Figure S8. The mass spectrum of *Bambusa tuldoides* (A) and papaverine (B).

A

B

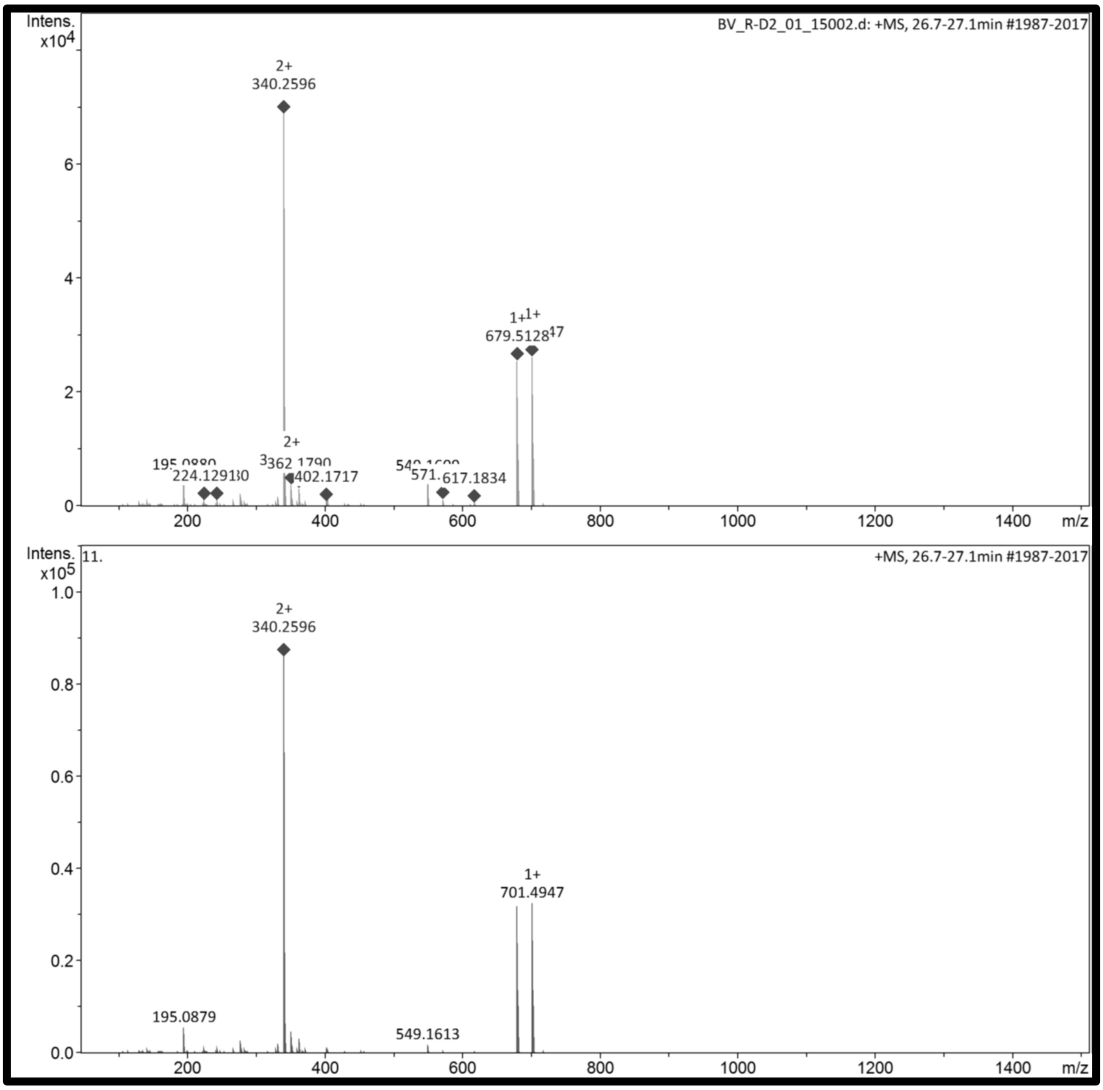

Figure S9. The mass spectrum of *Bambusa vulgaris* (A) and papaverine (B).

A

B

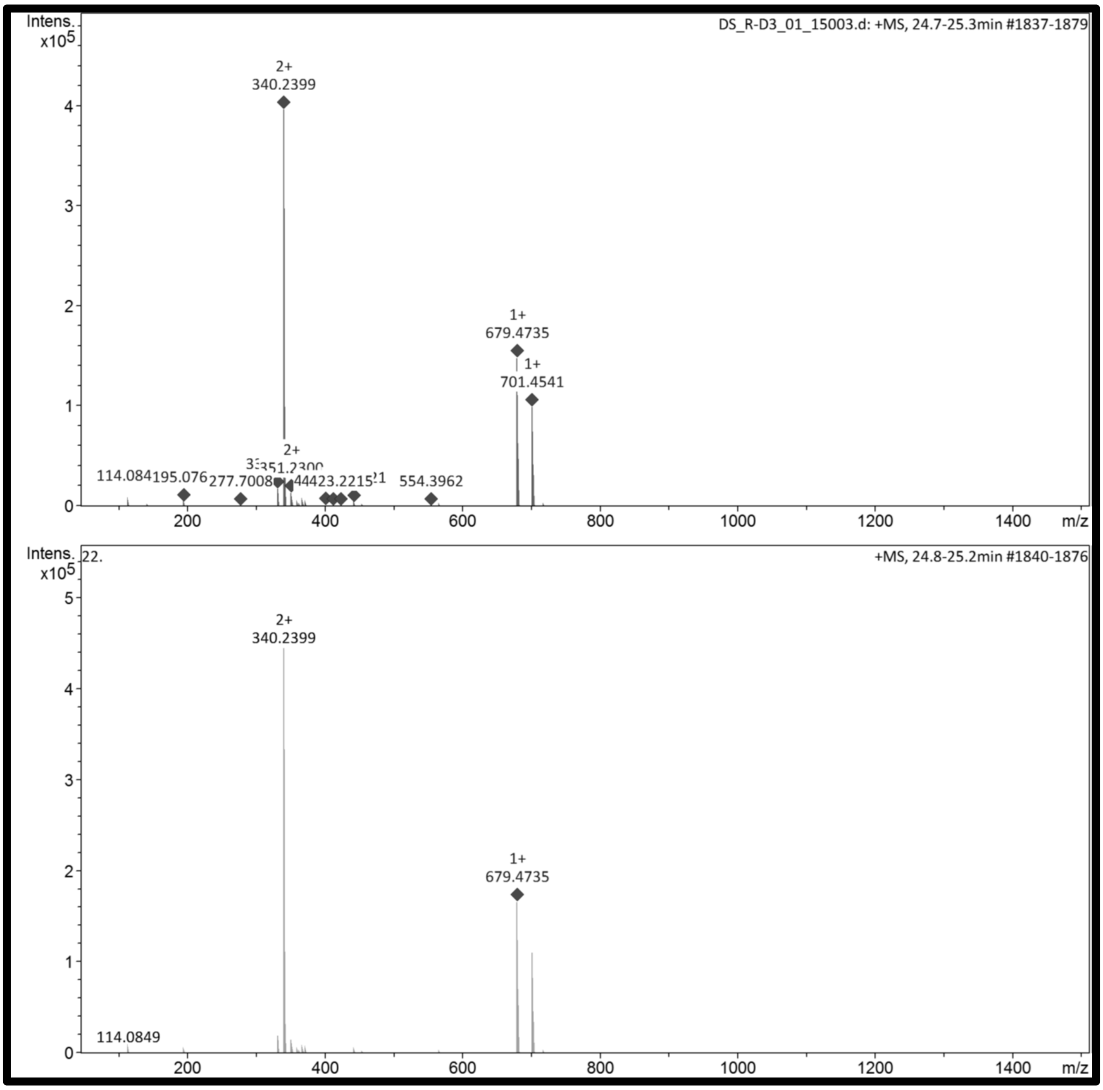

Figure S10. The mass spectrum of *Dinochloa sublaevigata* (A) and papaverine (B).

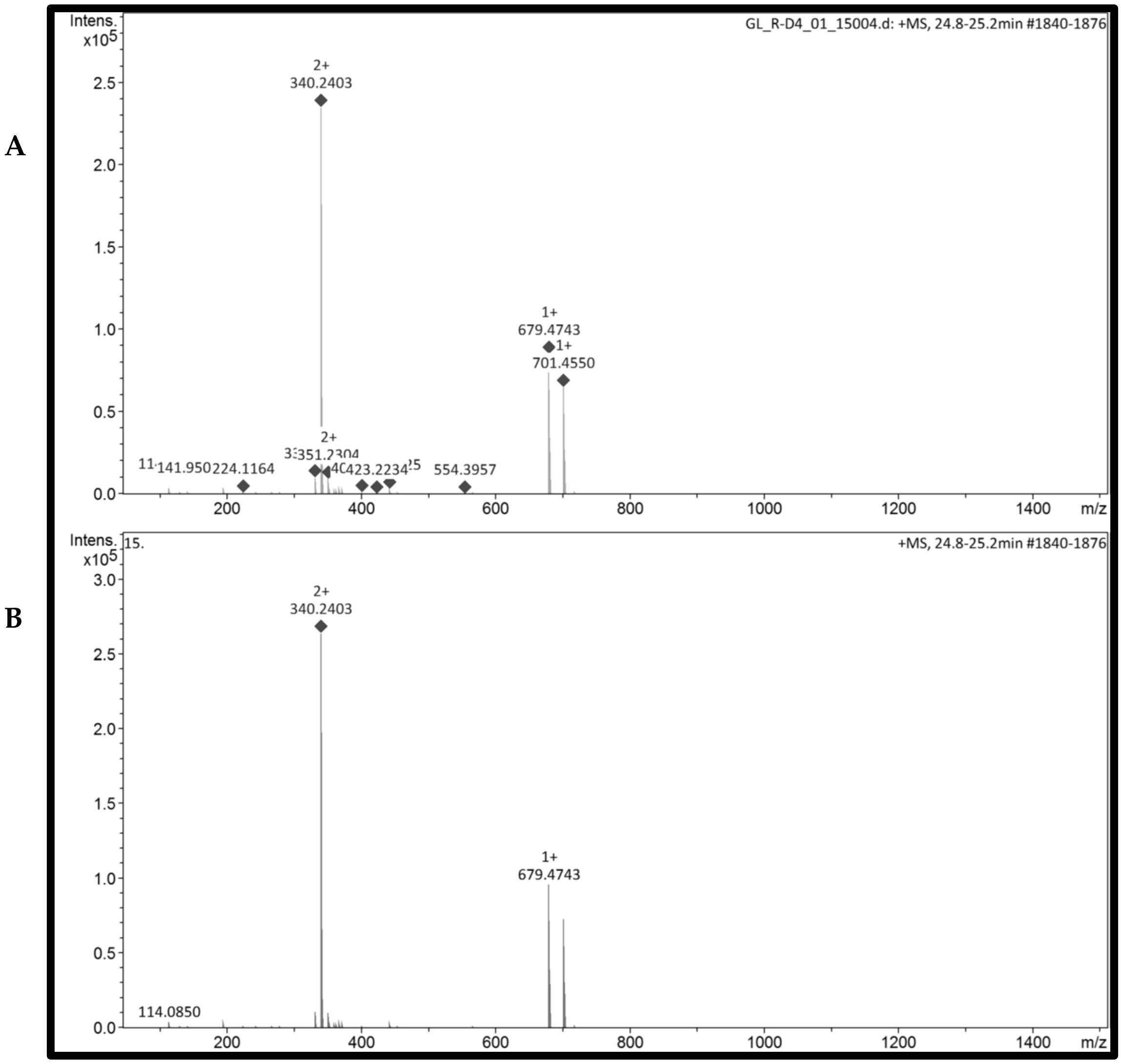

Figure S11. The mass spectrum of *Gigantochloa levis* (A) and papaverine (B).
